# Reliability and validity of the Turkish version of Post-COVID-19 Functional Status Scale

**DOI:** 10.3906/sag-2105-125

**Published:** 2021-10-21

**Authors:** Ebru ÇALIK KÜTÜKCÜ, Aslıhan ÇAKMAK, Esra KINACI, Oğuz Abdullah UYAROĞLU, Naciye VARDAR YAĞLI, Gülay SAİN GÜVEN, Melda SAĞLAM, Lale ÖZIŞIK, Nursel ÇALIK BAŞARAN, Deniz İNAL İNCE

**Affiliations:** 1 Department of Physiotherapy and Rehabilitation, Faculty of Physical Therapy and Rehabilitation, Hacettepe University, Ankara Turkey; 2 Department of Internal Medicine, Faculty of Medicine, Hacettepe University, Ankara Turkey

**Keywords:** Covid 19, SARS-CoV-2 infection, functional status, dyspnea, reliabilities, test-retest

## Abstract

**Background/aim:**

The post-COVID-19 Functional Status (PCFS) has recently been developed for functional outcomes of COVID-19 upon discharge and in long term. The purpose of this study was to investigate the reliability and validity properties of the Turkish version of the PCFS in Turkish post-COVID-19 patients with hospitalized and nonhospitalized during infection.

**Materials and methods:**

One hundred participants with post-COVID-19 were included in this cross-sectional study. Test-retest reliability of the Turkish version of PCFS assessed by intraclass correlation coefficient (ICC) and Cronbach’s alpha was calculated for internal consistency. For construct validity, correlation coefficients between the Turkish version of PCFS developed by translation-back translation method and modified Medical Research Council (mMRC) dyspnea scale (MMRC), London Chest Activities of Daily Living (LCADL) scale, Barthel Index (BI) were analyzed.

**Results:**

For test-retest reliability analysis, ICC ranged between 0.734 and 0.880. The total ICC score was 0.821, indicating excellent reliability. The Cronbach’s alpha value of the PCFS test and retest scores were recorded as 0.821 indicating that the scale is quite reliable. The PCFS score was moderately correlated with the mMRC score (r = 0.534, p < 0.001) and weakly correlated with the LCADL self care (r = 0.311, p = 0.002), domestic (r = 0.277, p = 0.005), physical activity (r = 0.342, p < 0.001), leisure subscores (r = 0.434, p < 0.001) and total score (r = 0.399, p < 0.001).

**Conclusion:**

The Turkish version of the PCFS scale is reliable scale that reflects activity limitation and functional status after COVID-19. The Turkish version of the PCFS will be a guide for rehabilitation professionals to understand functional limitation after COVID-19 and to direct interventions accordingly to functional status of the patients at discharge and in long term.

## 1. Introduction

The new type of Coronavirus (SARS-CoV-2) pandemic puts a great pressure on health systems around the world [1]. Coronavirus Disease 2019 (COVID-19) has a wide range of clinical manifestations, ranging from an asymptomatic state or mild respiratory symptoms to severe viral pneumonia and acute respiratory distress syndrome (ARDS) [2].

The individuals who survived after ARDS were reported to have significantly lower exercise capacity and health status than the general population, even after two years [3]. Persistent physical, cognitive and psychosocial disorders can be seen in survivors of ARDS [1,3]. Vaes et al. reported that patients recovered after COVID-19 have still persistent COVID-19-associated symptoms, poor working capacity and health status, moderate-severe functional limitations at 6 months follow-up [4]. 

Given the clinical heterogeneity of COVID-19 and the large number of survivors of COVID-19 that require follow-up, it is important to have a simple tool for the disease to monitor the course of symptoms and the impact of symptoms on patients’ functional status. Klok et al. developed post-COVID-19 Functional Status (PCFS) scale to assess functional status to capture the heterogeneity of post-COVID-19 outcomes [5]. They reported that the PCFS could be used after discharge at 4 and 8 weeks to directly monitor recovery, and at 6 months to assess functional sequelae. The PCFS scale could was designed to be used as an additional outcome measure to evaluate the final consequences of COVID-19 on functional status, not to replace other relevant tools for measuring quality of life, fatigue or dyspnea in the acute phase. The PCFS score ranges between 0 and 5 in which 0 indicates no functional limitation and 5 indicates death [5]. The supporting information such as a manual and various translations of the PCFS scale are freely accessible via its website (https ://osf.io/qgpdv /(CC-BY 4.0)). 

However, only one study has investigated construct validity of the PCFS scale in individuals with post-COVID-19 [6]. There is no valid and reliable and disease-spesific tool to evaluate functional status after COVID-19 in the Turkish population. Therefore, the aim of this study is to investigate the test-retest reliability and construct validity of the Turkish version of PCFS scale in Turkish population. This tool will be a guide to understand functional limitations of patients after COVID-19 and ease selection of proper post-COVID-19 patients who can benefit from rehabilitation at discharge and during recovery period and to evaluate the efficacy of rehabilitation interventions for rehabilitation professionals. 

## 2. Materials and methods

The study was conducted between September 2020 and December 2020. The study was carried out at the Cardiopulmonary Rehabilitation Unit of Faculty of Physical Therapy and Rehabilitation, Hacettepe University. The sample size of the study was determined as 5 times of the number of items used in the scale [7]. We tried to reach as many participants as possible to increase the strength of the study. 

### 2.1. Participants

The inclusion criteria for participants were being ≥ 18 years, having an education level at least primary school, being a native Turkish speaker, being hospitalized or nonhospitalized post-COVID-19 patients in recovery period. The individuals who were hospitalized in the intensive care unit (ICU), have severe neuromuscular and musculoskeletal problems, have cognitive problems, who are unable to cooperate, unable to read and write, and not willing to participate were excluded. Hacettepe University Non-Interventional Clinical Research Ethics Committee approved the study on 01.09.2020, with the registration number GO 20/788. All participants were informed about the study protocol. This study was registered in ClinicalTrials.gov: NCT04584450.

### 2.2. Translation and cultural adaptation

First of all, the permission to investigate the construct validity and test-retest reliability of PCFS scale in the Turkish population was obtained from the developer of the PCFS scale. The PCFS scale was translated by two native Turkish speakers who have a good command of English. A common version was formed with the synthesis of the two translations. Two independent native English speaker proficient in Turkish who had not studied on the first translation process performed retranslation (from Turkish to English). The original and re-translated versions of the PCFS scale were compared and reviewed by the expert committee that consists three physiotherapists, and the prefinal version was created. A pilot group of 30 patients with post-COVID-19 assessed the understandability of the scale and gave their inputs. After the pilot group assesments completed, the final form of the PCFS scale was given by the committee based on the findings [8].

### 2.3. Evaluations

The participants were asked to fill out an online form using Google Forms. The physical characteristics, smoking history, symptoms, comorbidities, the length of stay in hospital and time since first COVID-19 diagnosis were recorded. The diagnosis of COVID-19 was confirmed based on reverse transcription polymerase chain reaction (RT-PCR) test and/or computed tomography (CT) scan of the thorax. For test-retest reliability, the questionnaire was repeated twice at an interval of 7 days. 

The PCFS scale stratifies functional status limitation as follows: grade 0 (No functional limitations), grade 1 (Negligible functional limitations), grade 2 (Slight functional limitations), grade 3 (Moderate functional limitations), grade 4 (Severe functional limitations), and grade 5 (death) [5]. 

Perceived functional limitations during daily life as a result of dyspnea was assessed using the modified Medical Research Council (mMRC) dyspnea scale. Individuals are asked to choose the expression that best describes their dyspnea level. The scoring in mMRC varies between 0–4 points. “0 points” means that there is no dyspnea, and “4 points” indicates that there is a perception of dyspnea during basic daily life activities such as dressing [9].

The Turkish version of the London Chest Activities of Daily Living (LCADL) Scale was used to evaluate the construct validity of the PCFS scale [10]. The LCADL scale consists of 15 items and four components: self-care (4 items), domestic (6 items), physical activity (2 items), and leisure (3 items). Each item is scored between 0 and 5. High scores show that the limitation in daily living activities due to dyspnea symptom is greater. The maximal score that can be reached is 75 [10].

The Turkish version of the Barthel Index (BI) was also used to the construct validity of the PCFS scale. This simple and understandable BI consists of 10 subheadings: Feeding, bathing, self-care, dressing, bladder control, bowel control, toilet use, chair/bed transfer, mobility, use of stairs. The total score ranges from 0 to 100. The higher score reflects a greater ability to function independently following hospital discharge. The BI score could be classified as follows: 0–20 indicates “total” dependency, 21–60 indicates “severe” dependency, 61-90 indicates “moderate” dependency, and 91–99 indicates “slight” dependency [11].

### 2.4. Statistical analysis

The statistical analysis was performed using the SPSS for Windows (Version 23.0, IBM Inc., Armonk, NY, USA). The data were expressed as mean±standard deviation (SD) and minimum-maximum for quantitative variables and as percentage (%) for categorical variables. 

The construct validity of the PCFS scale was measured using correlation coefficients between the Turkish version of PCFS scale and mMRC dyspnea scale, LCADL scale, BI. The internal consistency of the PCFS scale was assessed using Cronbach’s α coefficient. A cronbach’s alpha value 0.60 ≤ α ≤ 0.79 is considered quite reliable and α ≥ 0.80 is considered highly reliable [12]. The test-retest reliability was measured using the intraclass correlation coefficients (ICC). ICC values ranges from 0.00 to 1.00, with values of 0.60 to 0.80 demonstrates good reliability and ICC values above 0.80 indicates excellent reliability [13]. The relationships between the parameters were analyzed assessed using Spearman’s rank correlation coefficients accordingly to the normality. The correlation coefficient was interpreted as little or no (0 to 0.25), weak (0.26 to 0.49), moderate (0.50 and 0.69), strong (0.70 and 0.89), very strong (0.90 and 1.00) [14]. The probability of error in the statistical analyses was determined as p < 0.05 [15]. 

## 3. Results

One hundred individuals (mean age = 36.6±13.8 years, female/male = 59/41) were included in the study. The physical characteristics, smoking status, symptoms, comorbidities, and the length of stay in hospital were shown in Table 1. Sixty percentage of post-COVID-19 patients were hospitalized (without admission to the ICU), and 40% of patients were nonhospitalized during infection period. Whereas, most of the post-COVID-19 patients (43%) reported no functional limitation, 31% of patients had grade 1, 20% of patients had grade 2, and 6% of patients had grade 3 functional limitation according to PCFS. 

**Table 1:** The clinical characteristics of the participants.

Parameters	COVID-19 Survivors (n = 100)	
	Mean±SD	Min-Max
Age (years)	36.6 ± 13.8	18-82
Sex (female/male), n	59/41	
Weight (kg)	69.8 ± 15.5	47–188
Height (cm)	168.9 ± 9.1	153–200
BMI (kg/m2)	24.4 ± 4.5	11.5–37.2
Smoking (pack-years)	3.4 ± 7.6	0–35
	n	%
Smoking history		
Smoker	9	9
Ex-smoker	22	22
Non-smoker	69	69
Symptom perceptions		
Resting dyspnea	4	4
Effort dyspnea	58	58
PND	6	6
Ortopnea	8	8
Cough	20	20
Sputum	23	23
Education level		
Literate	2	2
Primary school	5	5
Middle school	8	8
High school	21	21
University	49	49
Higher degree	15	15
Marital Status		
Married	59	59
Single	36	36
Divorced	5	5
Working Status		
Unemployed	5	5
Student	14	14
Retired	11	11
Full time work	57	57
Part time work	3	3
Housewife	10	10
	Median	Min-Max
mMRC dyspnea score (0-4)	1	0–3
CCI score	0	0–7
	Mean±SD	Min-Max
Length of stay (days)	4.5 ± 7.0	0–54
Time since COVID-19 diagnosis (months)	2.7 ± 1.5	1–6
LCADL		
LCADL-self-care score	4.3 ± 1.0	2–9
LCADL-domestic score	6.1 ± 3.6	0–22
LCADL-physical activity score	2.9 ± 1.3	1–7
LCADL-leisure score	3.6 ± 1.2	2–9
LCADL-total score	17.0 ± 5.7	6–40
The degree dyspnea perception affects daily life in general	n	%
A lot/a little/not at all	7/45/48	7/45/48
Barthel ADL Index		
Barthel ADL Index total score (0-100)	96.9 ± 12.2	0–100
Barthel functional classification	n	%
Independent	82	82
Slight dependency	12	12
Moderate dependency	4	4
Severe dependency	1	1
Total dependency	1	1

Abbreviations: SD: standard deviation, BMI: Body Mass Index, CCI: Charlson Comorbidity Index, mMRC: Modified Medical Research Council Dyspnea Scale, PND: Paroxysmal nocturnal dyspnea.

### 3.1. Internal consistency and test-retest reliability 

Internal consistency and test-retest reliability of the PCFS scale were shown in Table 2. The Cronbach’s alpha value of the PCFS test and retest scores were recorded as 0.821 indicating that the scale is highly reliable (Table 2). The ICC values ranged from 0.734 to 0.880 (Table 2). According to the mean ICC value, the PCFS test-retest reliability results were excellent. The PCFS test score was also significantly correlated with PCFS retest score (r = 0.707, p < 0.001, Table 3). 

**Table 2:** The internal consistency and intraclass correlation (ICC) coefficients values of the PCFS.

	1st TestMedian (Min-Max)	2nd TestMedian (Min-Max)	
PCFS score	1 (0–3)	1 (0–3)	
	Cronbach’s α	ICC	95% CI
PCFS score	0.821	0.821	0.734–0.880

Abbreviations: PCFS: Post-COVID-19 Functional Status Scale, ICC: Intraclass correlation coefficient; CI: Confidence interval.

**Table 3:** The bivariate correlations between the PCFS score and scores of the criterion scales.

Parameters	COVID-19 Survivors(n = 100)	
	PCFS score	
	r	p
PCFS test-retest score	0.707	< 0.001*
mMRC dyspnea score (0–4)	0.534	< 0.001*
LCADL	0.311	0.002*
Self-care score	0.277	0.005*
Domestic score	0.342	< 0.001*
Physical activity score	0.434	< 0.001*
Leisure score	0.399	< 0.001*
Barthel Index total score (0–100)	0.095	0.348

Abbreviations: PCFS: Post-COVID-19 Functional Status Scale, mMRC: Modified Medical Research Council Dyspnea Scale, LCADL: London Chest Activity of Daily Life Scale.*p < 0.05, Spearman correlation analysis.

### 3.2. Validity

The correlation coefficients between the PCFS score and the criterion questionnaires are presented in Table 3. The PCFS score was moderately correlated with the mMRC dyspnea scale (r = 0.534, p < 0.001) and weakly correlated with the LCADL self care (r = 0.311, p = 0.002), domestic (r = 0.277, p = 0.005), physical activity (r = 0.342, p < 0.001), leisure subscores (r = 0.434, p < 0.001), and total score (r = 0.399, p < 0.001). There was not any association between the scores of PCFS and BI (p>0.05, Table 3). 

## 4. Discussion

This study demonstrated the Turkish version of PCFS is reliable and has high internal consistency. The PCFS scale has moderate relation with the functional status measures that reflects activity limitation and daily physical activity level. 

To the best of our knowledge, there is no published reliability and validity studies of the PCFS for any languagein the literature. The present study demonstrated that the Turkish version of the PCFS scale has high internal consistency level with Cronbach’s alpha value (0.821). This shows us that the PCFS scores are stable over time despite one week time interval between test and retest. We also found high ICC values for test-retest reliability and strong correlation between test and retest PCFS scores in our study. An excellent level of reliability of the measuring tool is a quality indicator for this tool. We think that the Turkish version of the PCFS scale has a high level of reliability for evaluating functional status after COVID-19 infection.

The developers of the PCFS scale reported that this scale can be used for assessing functional status after discharge and for long term functional results after COVID-19. They also stated that the usefulness of the PCFS scale depends on the local conditions [5]. Despite there is no published reliability studies of the PCFS scale in any language, the construct validity of the PCFS scale was demonstrated very recently in highly-symptomatic post-COVID-19 patients three months after the onset of symptoms [6]. For construct validity, we assessed the correlations between the PCFS score and the scores of mMRC dyspnea scale, LCADL and BI. We especially selected the mMRC dyspnea and LCADL as indicators of activity limitation related with dyspnea [16]. We used the BI as a measure of functional performance in ADL [11]. We selected BI for construct validity because BI was one of the most used assessment tools for evaluating ADL in post-COVID-19 [17] and BI also gives opportunity for assessing all parameters of daily life activities [11]. We demonstrated the Spearman correlation coefficient value of the PCFS score with the mMRC dyspnea scale score was 0.534. Furthermore, the PCFS score was weakly correlated with the LCADL self care (r = 0.311), domestic (r = 0.277), physical activity (r = 0.342), leisure subscores (r = 0.434) and total score (r = 0.399). The most of post-COVID-19 patients (>70%) with mean PCFS score (2.3±1.1 for hospitalized patients, 2.4±0.8 for non-hospitalized patients) were reported to have dyspnea symptom 3 months after infection [4]. Another study confirmed that as the PCFS score increases, there is gradual increase in presence and severity of symptoms, decreased work productivity, daily usual activities and poorer quality of life in post-COVID-19 patients after three months [6]. We found that the Turkish version of PCFS scale was moderately correlated with the MMRC scale but weakly correlated with the LCADL scale. The moderate association between the MMRC scale score and the PCFS score can be related with that 58% of post-COVID-19 patients have effort dyspnea during daily life and both scale is a functional limitation grading system [5]. The mMRC dyspnea was also shown to be a predictor of low physical activity level [18]. The significant relationship between the mMRC dyspnea and PCFS scales can be a result of that the PCFS scale evaluates functional limitations including changes in lifestyle, sports and social activities [5]. It is common aspect for both scales to reflect limitations in physical activity and daily living activities. This close relation also confirms the previous findings that persistent dyspnea limits ADL during follow-up in post-COVID-19 patients [4, 6]. The PCFS scale also concerns functional limitations related with the symptoms like the dyspnoea, pain, fatigue, muscle weakness, memory loss, depression, and anxiety related with the COVID-19 [5]. This weak correlation between the PCFS score and LCADL total and subscores can be expected since the LCADL scale is predominantly concerned with dyspnea, whereas the PCFS scale investigates other symptoms such as pain, fatigue, and muscle weakness [7,10]. The BI is generally used to assess functional limitation in daily life especially due to neurological disorders [19]. The BI were also used in respiratory diseases and can demonstrate the functional effect of disease on daily living activities in geriatric respiratory diseases [20]. Although the literature showed that the BI could be able to determine ADL limitation in 60% of individuals after COVID-19 [17], the mean score was 90 after COVID-19 discharge in patients without ventilation support during hospital [21]. The need for help for personal care also significantly decreases from discharge to 6 month follow-up in post-COVID-19 patients [4]. The mean BI score of our post-COVID-19 patients that mainly consist of patients followed at home and hospital without ICU admission was also above 90, and only two patients had severe or total dependency during daily living activities. Otherwise, we think that the reason for any association between the PCFS score and the BI total score could be that the BI may have underestimated real physical limitation for not considering dyspnea, fatigue, pain, or depression/anxiety [11,19]. This index evaluates only dependency and does not consider the effects of symptoms on daily life activities, unlike the PCFS. So, this could lead to any association between the two measures. 

The main limitation of our study was that we didn’t include patients with COVID-19 who required ICU admission. This may lead to no association between the PCFS score and BI total score because any participants stated severe dependency (score 4) on the PCFS scale. According to our knowledge, the strength of this study is being the first study that investigates psychometric properties of the Turkish version of the PCFS scale. 

In conclusion, the Turkish version of the Post-COVID-19 Functional Status Scale with excellent reliability can be used for evaluating functional status of Turkish patients with post-COVID-19. This simple, useful and inexpensive tool is also closely related with functional status measures that evaluates the effect of dyspnea, which is one of the main complaint of individuals with post-COVID-19 on activity limitation. 

## Informed consent

The study was approved by the Hacettepe University Non-Interventional Clinical Research Ethics Committee (Date: 01.09.2020, Number: GO 20/788). Each participant gave consent to participate.

## References

[ref1] Simpson R 2020 Rehabilitation after critical illness in people with COVID-19 infection American Journal of Physical Medicine and Rehabilitation 99 470 474 32282359 10.1097/PHM.0000000000001443 PMC7253039

[ref2] Mohamed AA Alawna M. 2020 Role of increasing the aerobic capacity on improving the function of immune and respiratory systems in patients with coronavirus (COVID-19): a review Diabetes&Metabolic Syndrome 14 489 496 32388326 10.1016/j.dsx.2020.04.038 PMC7186129

[ref3] Ngai JC Ko FW Ng SS To KW 2010 Tong M et al Respirology 15 543 550 20337995 10.1111/j.1440-1843.2010.01720.x PMC7192220

[ref4] Vaes AW Goërtz YMJ Van Herck M Machado FVC Meys R 2021 Recovery from COVID-19: a sprint or marathon? 6 months follow-up data of online long COVID-19 support group members ERJ Open Research 7 00141 2021 34041295 10.1183/23120541.00141-2021 PMC8012818

[ref5] Klok FA Boon GJAM Barco S Geelhoedet JJM 2020 The Post-COVID-19 Functional Status Scale: a tool to measure functional status over time after COVID-19. European Respiratory Journal 56 2001494 2001494 10.1183/13993003.01494-2020 PMC7236834 32398306

[ref6] Machado FVC Meys R Delbressine JM Vaes AW 2021 Goërtz YMJ et al Health Quality Life Outcomes 19 40 40 10.1186/s12955-021-01691-2 PMC7856622 33536042

[ref7] Tsang S Royse CF Terkawi AS 2017 Guidelines for developing, translating, and validating a questionnaire in perioperative and pain medicine Saudi Journal of Anaesthesia 11 S80 S89 28616007 10.4103/sja.SJA_203_17 PMC5463570

[ref8] Beaton D Bombardier C Guillemin F Ferraz MB 2000 Guidelines for the process of cross-cultural adaptation of self-report measures Spine 25 3186 3191 11124735 10.1097/00007632-200012150-00014

[ref9] Crisafulli E Clini EM 2010 Measures of dyspnea in pulmonary rehabilitation Multidisciplinary Respiratory Medicine 5 202 210 22958431 10.1186/2049-6958-5-3-202 PMC3463047

[ref10] Saka S Savci S Calik-Kutukcu E Saglam M Vardar-Yagli N 2020 Validity and reliability of the Turkish version of the London Chest Activity of Daily Living Scale in obstructive lung diseases Turkish Thoracic Journal 21 116 121 32203002 10.5152/TurkThoracJ.2019.18155 PMC7089701

[ref11] Kucukdeveci AA Yavuzer G Tennant A Suldur N 2000 Sonel B et al Scandinavian Journal of Rehabilitation Medicine 32 87 92 10853723

[ref12] Alpar R. Spor 2020 Sağlık ve Eğitim Bilimlerinden Örneklerle Uygulamalı İstatistik ve Geçerlik-Güvenirlik

[ref13] Weir JP 2005 Quantifying test-retest reliability using the intraclass correlation coefficient and the SEM The Journal of Strength and Conditioning Research 19 231 240 15705040 10.1519/15184.1

[ref14] Plichta SB Kelvin EA 2013 Munro’s Statistical Methods for Health Care Research China: Lippincott Williams & Wilkins;

[ref15] Hayran M Hayran M. Sağlık Araştırmaları İçin Temel İstatistik 2011

[ref16] Lareau SC Blackstock FC. 2019 Functional status measures for the COPD patient: A practical categorization Chronic Respiratory Disease 16 1479973118816464 1479973118816464 30789020 10.1177/1479973118816464 PMC6318724

[ref17] Pizarro-Pennarolli C Sánchez-Rojas C Torres-Castro R Vera-Uribe R Sanchez-Ramirez DC 2021 Assessment of activities of daily living in patients post COVID-19: a systematic review Peer Journal 9 10.7717/peerj.11026 PMC8034364 33868804

[ref18] Hayata A Minakata Y Matsunaga K N. 2016 Differences in physical activity according to mMRC grade in patients with COPD International Journal of Chronic Obstructive Pulmonary Disease 11 2203 2208 27695306 10.2147/COPD.S109694 PMC5028078

[ref19] Taghizadeh G Martinez-Martin P Meimandi M Habibi SAH Jamali S 2020 Barthel Index and modified Rankin Scale: Psychometric properties during medication phases in idiopathic Parkinson disease Annals of Physical and Rehabilitation Medicine 63 500 504 31816448 10.1016/j.rehab.2019.08.006

[ref20] Peruzza S Sergi G Vianello A Pisent C Tiozzo 2003 Chronic obstructive pulmonary disease (COPD) in elderly subjects: impact on functional status and quality of life Respiratory Medicine 97 612 617 12814144 10.1053/rmed.2003.1488

[ref21] Sakai T Hoshino C Yamaguchi R Hirao M Nakahara R 2020 Remote rehabilitation for patients with COVID-19 Journal of Rehabilitation Medicine 52 16501977 2731 10.2340/16501977-2731 32871014

